# An extended machine learning technique for polycystic ovary syndrome detection using ovary ultrasound image

**DOI:** 10.1038/s41598-022-21724-0

**Published:** 2022-10-12

**Authors:** Sayma Alam Suha, Muhammad Nazrul Islam

**Affiliations:** Military Institute of Science and Technology, Department of Computer Science and Technology, Dhaka, 1216 Bangladesh

**Keywords:** Computational biology and bioinformatics, Health care

## Abstract

Polycystic ovary syndrome (PCOS) is the most prevalent endocrinological abnormality and one of the primary causes of anovulatory infertility in women globally. The detection of multiple cysts using ovary ultrasonograpgy (USG) scans is one of the most reliable approach for making an accurate diagnosis of PCOS and creating an appropriate treatment plan to heal the patients with this syndrome. Instead of depending on error-prone manual identification, an intelligent computer-aided cyst detection system can be a viable approach. Therefore, in this research, an extended machine learning classification technique for PCOS prediction has been proposed, trained and tested over 594 ovary USG images; where the Convolutional Neural Network (CNN) incorporating different state-of-the-art techniques and transfer learning has been employed for feature extraction from the images; and then stacking ensemble machine learning technique using conventional models as base learners and bagging or boosting ensemble model as meta-learner have been used on that reduced feature set to classify between PCOS and non-PCOS ovaries. The proposed technique significantly enhances the accuracy while also reducing training execution time comparing with the other existing ML based techniques. Again, following the proposed extended technique, the best performing results are obtained by incorporating the “VGGNet16” pre-trained model with CNN architecture as feature extractor and then stacking ensemble model with the meta-learner being “XGBoost” model as image classifier with an accuracy of 99.89% for classification.

## Introduction

Polycystic ovary syndrome (PCOS) is one of the most widespread endocrinological anomalies affecting one out of every ten pre-menopausal reproductive women worldwide. PCOS is associated with excessive rise in male androgen hormone in the female body, which causes a persistent disruption in hormonal levels and as a consequence, adversely affects normal ovarian functions, resulting in the growth of numerous cysts inside the ovary^1^. PCOS has been recognized as the leading cause of anovulatory infertility as well as being related to a range of metabolic and psychological disorders; including irregular menstrual periods, hirsutism, abrupt obesity, type 2 diabetes, thyroid abnormalities, increased depression, sexual dissatisfaction, etc. lowering the quality of a healthy way of life^2,3^. Multiple studies have also revealed that, women having PCOS are at higher risk of suffering from endometrial and ovarian cancer which can lead to death if not detected early^4,5^. However, accumulating evidence suggests that if a well-standardized diagnostic approach can be used to identify PCOS in a timely manner, the condition can be recovered by appropriate, symptom-oriented, long-term and dynamic treatments^6^.

The Rotterdam criteria for polycystic ovarian syndrome (PCOS) are three criteria that are commonly used to diagnose PCOS by a wide spectrum of medical practitioners; those are: hyperandrogenism, menstrual irregulations and existance of multiple cysts in ovary ultrasonography^7^. Among these, the identification of numerous cysts using ultrasound scanning is the most reliable method of detecting PCOS^8^. But, because of the reliance on the observer and the presence of significant noise in these images, medical analysis can be time-consuming and difficult, with the risk of human mistake. In addition, in least developed and underdeveloped countries, there is a scarcity of experienced radiologists who can appropriately diagnose ovarian ultrasound. As a result, many young women who are suffering from this serious condition go undetected and untreated for long periods of time. Therefore, researchers worldwide are now working to develop effective PCOS detection approach that would employ a variety of modern computational techniques.

However, the typical methodologies applied for detecting PCOS using computational approaches rely on several image processing techniques for feature extraction and then traditional machine learning strategies for image classification which is a tedious process with relatively lower performances. Again, some researchers also performed deep learning approaches to detect PCOS from ultrasound images using Convolutional Neural Network(CNN). Though deep learning algorithms typically attain a high level of accuracy in categorizing images, they have the limitation of consuming a lot of computing complexity and time to execute which becomes a barrier to utilize them in practical applications^9^. As such, an integrated or extended ML based approach may enhance the prediction performance and reduce the computational complexity to predict PCOS using image data.

Therefore, the objective of this research is to propose an extended machine learning classification technique for PCOS prediction using ovary USG images. The significant contributions that has been conducted to achieve the goal of this study are listed below.

- A deep learning method incorporating different state-of-the-art techniques like transfer learning with several pre-trained models in a CNN architecture has been utilized for feature extraction from the input ultrasound ovary images. And then a stacking ensemble machine learning model employing five traditional models as base learners with one boosting or bagging ensemble model as meta-learner, have been employed on that reduced feature set to classify them into PCOS or non-PCOS criterion.
- For inspecting the performance of the proposed methodology, four different types of machine learning techniques have been trained and tested with same dataset for PCOS detection, including the conventional machine learning technique; conventional machine learning technique with feature reduction; deep learning technique, and the proposed extended technique.
- Multiple ablation studies have been conducted to analyze the impact of different stages of machine learning such as image pre-processing in case of performing conventional machine learning technique; transfer learning in case of executing deep learning technique. Furthermore, the outcomes of various approaches employing several types of machine learning models are examined through relevant performance metrics in order to determine the optimal strategy for PCOS ultrasound image categorization.

The rest of the paper is organized as follows: the related works are presented in “Related works” section; and the methodology applied in this study is discussed in “Methodology” section; “Exploring machine learning techniques” section represents the result analysis with comparative findings; and finally, “Result analysis” section includes discussion and conclusion that summarizes the key findings, limitations, and future plan for the research.

## Related works

The most prominent practice used by physicians to accurately diagnose PCOS is to examine ultrasound images of the ovaries to examine for the existence of multiple follicular cysts and hence determine whether or not the patient has PCOS^10^. Ultrasound imaging is a well-known diagnostic technology that uses ultrasound waves generated by a transducer to create images of human body parts that provide real-time, precise anatomical and physiological information^11^. In the case of ovarian ultrasound imaging, the ultrasonographers or radiologists visually assess the obtained ultrasound pictures for any abnormalities in the ovaries, and if several cysts with higher measurements of diameters are observed, then PCOS is diagnosed^12^. Despite the fact that this is the most usual method to diagnose ovarian abnormality, the correctness and reliability of visual interpretations are frequently dependent on the observer’s expertise and also several types of noises have an impact on ultrasound imaging making it harder for observers to diagnose^13^. Therefore, to replace this arduous, error-prone, and time-consuming manual method of PCOS diagnosis; several researchers worldwide are exploring computer-assisted techniques of follicle identification and diagnosis of PCOS, which provide significant benefits such as rapid ultrasound image processing in the quickest time frame with reduced diagnostic mistake and human involvement^14^.

For developing computer-aided PCOS follicle detection system, the implementation of various forms of digital image processing techniques is the most frequently utilized strategy. For example, Mandal et al.^15^ suggested a technique to diagnose PCOS by automatically segmenting cysts and follicle regions from ultrasound images; for which they employed multiple digital image processing steps such as histogram equalization, K-means clustering, median filtering, and morphological erosion on 19 ultrasound pictures. Yilmaz et al.^16^ tested and compared two methods of follicle detection using image processing techniques to diagnose PCOS; where the first approach includes noise reduction (Median, Average, Gaussian, and Wiener Filters), contrast modification (histogram equalization and adaptive thresholding), binarization, and morphological procedures.; and the second approach comprises of noise reduction (Gaussian Filter and Wavelet Transform), k-means clustering, hole filling and morphological operations. Further, they compared the analysis results using two performance metrics : False Acceptance Rate (FAR) and False Rejection Rate (FRR). Gopalakrishnan et al.^17^ have suggested an image processing-based strategy for PCOS detection utilizing the combination of modified Otsu method with active contour to determine the precise quantity of cysts from the ultrasound ovary image; their proposed method contains mainly two parts that is image pre-processing and follicle identification. In pre-processing stage they performed Region of Interest (ROI) extraction, speckle noise reduction using various strategies; then exploring the best performing filter technique they used the modified Otsu approach with active contour method to accomplish the second phase of follicle identification, which included image segmentation and feature extraction. Setiawati et al.^18^ suggested a clustering approach that can be utilized for PCOS detection employing Particle Swarm Optimization (PSO) technique with a non-parametric fitness function to create more compacted and converging clusters for follicular segmentation.Sitheswaran et al.^19^ used object growing methodology to detect PCOS in two stages; where first step pre-processing comprised median filtering, local maximum extraction with ROI selection, and the second stage of follicle detection included cost map development, which finally provided a convex hull of probable follicles to decide object growing. Mehrotra et al.^20^ proposed another method where the input ultrasound picture would be preprocessed for noise reduction and contrast enhancement using a multiscale morphological technique and then the follicles would be segmented using scanline thresholding; they also conducted a comparative analysis of the manual result with the acquired result to assess its effectiveness. Again, Deng et al.^21^ suggested another automated scheme in which an adaptive morphological filter was used to filter the input ovary ultrasound picture, then for contour extraction a modified labeled watershed algorithm was employed and lastly, to detect PCOS, a clustering algorithm was used to locate anticipated follicular cysts. Thus, these investigations mostly used image processing to locate follicles in ovarian USG pictures, but they seldom classified the images into PCOS or non-PCOS criteria.

Several researchers combined machine learning models with digital image processing techniques to develop an automated machine learning based PCOS detection system which will not only detect follicles but also will classify them into PCOS or non-PCOS classes. For example, Rachana et al.^22^ performed image enhancement, histogram equalization, Otsu thresholding, binarization, noise reduction, segmentation, feature extraction, and other relevant phases of image processing; and after that they used the KNN machine learning classification algorithm to categorize 50 USG photos. Nilofer et al.^23^ proposed a method where the images were first preprocessed using noise removal with median filter, segmentation using k-means clustering algorithm, feature extraction using GLCM method; and then a hybrid technique with artificial neural network (ANN) incorporating Improved Fruit Fly Optimization (IFFOA) was utilized to classify the images. On 65 ovary USG images, Gopalakrishnan et al.^24^ employed multiple conventional image processing approaches, including image enhancement, thresholding, noise reduction, Canny edge detection method to detect the follicle edge, and Scale-Invariant Feature Transform method to extract essential features; and furthermore they were classified using the Support Vector Machine (SVM), Decision Tree, and Naive Bayes classification algorithms, with SVM outperforming other models with 94.40% accuracy. Purnama et al.^25^ suggested another approach for PCOS identification, in which USG images are first preprocessed through various phases to create binary images, which are then segmented using edge detection, labeling, and cropping; later, based on the feature vectors resulted from feature extraction step using Gabor wavelet, 3 types of classification algorithm was implemented with SVM-RBF kernel classification method achieving the highest accuracy of 82.55%. Deshpande et al.^26^ suggested another strategy, in which they used image processing techniques like contrast enhancement, filtering, feature extraction using Multiscale morphological approach and segmentation to determine the number of follicles in ovarian ultrasound pictures; following that, integrating the number of follicles acquired from the image processing stage with other features of that patient such as body mass index (BMI), hormone levels etc. they applied Support Vector Machine classification algorithm to detect PCOS.

Furthermor, Deep learning (DL) strategies have nowadays gained a lot of momentum in clinical diagnosis using medical images since they have the advantages over conventional machine learning approaches to bypass the feature extraction and traditional image processing steps as the neural network itself conduct these tasks^27–29^. A few researchers recently have also utilized deep learning methods for PCOS detection. For example, Vikas et al.^30^ proposed a deep learning method for detecting PCOS, in which they initially implemented a three-layer Convolutional Neural Network (CNN) for detecting PCOS, then enhanced the model’s accuracy by incorporating data augmentation method, and finally obtained the best accuracy by using transfer learning (VGG16 pretrained model) with fine-tuning. Cahyono et al.^31^ suggested a Convolutional Neural Network (CNN) having 6 layers to classify 40 PCOS and 14 non-PCOS USG images, with the feature extraction phase done automatically using deep learning.; they employed the softmax activation function and tested the model’s performance using several alternative dropout and learning rates, with the best F1-score on test data being 76.36%.

Therefore, according to the previous studies PCOS and non-PCOS usg image identification has been so far conducted in two different ways: one group used image processing techniques to perform picture segmentation with feature extraction, and then a machine learning model to categorize the pre-processed images ; another group had applied deep learning methods to detect PCOS from USG images avoiding the image processing steps. Even though the PCOS detection had been conducted employing these techniques, but The follicular segmentation utilizing digital image processing requires cautious execution of several image processing stages. In addition, the extraction method using digital image processing techniques need to be very carefully tuned based on the varied picture quality and formats, which may need extra computing complexity and time, and thus each step becomes quite laborious. Also, it has been observed that employing digital image processing technique for feature extraction and then using conventional machine learning classifiers comparatively provides less accuracy in comparison to the deep learning methodologies. Also, a relatively small number of studies that used solely deep learning to diagnose PCOS encountered other difficulties, such as the prolonged processing times and high computational power necessary to handle the enormous amount of pictures needed to detect PCOS and thus it may become quite challenging to implement a practically usable interface for patients or medical professional using these deep learning approaches.

However, from the earlier studies it can be observed that, though there are number of contributions suggested from researchers across the world where several machine learning stretegies had been applied to detect PCOS; rarely has any researcher investigated the possibilities and efficacy of employing several types of ensemble machine learning techniques (bagging, boosting, and stacking) in this context. Ensemble techniques are a potential state-of-the-art solution for various machine learning difficulties, since they may considerably enhance a single model’s forecasting performance by training multiple models and integrating their predictions^32^. Recently, several researchers have successfully employed ensemble machine learning strategies in other fields of healthcare predictions. For example, Jabbar et al.^33^ proposed an ensemble learning strategy to solve the challenge of breast cancer data categorization; Kaur et al.^34^ applied stacking ensemble machine learning technique with an aim to identify brain tumor using Magnetic Resonance Imaging(MRI) of brain etc. Also, in a few recent research of other healthcare fields, the strengths of the CNN technique for feature extraction and the traditional machine learning technique for classification have been coupled to categorize medical images. For example, Pang et al.^35^ developed a technique of protein subcellular localization for Alzheimer’s disease prediction by using CNN as a feature extractor to automatically extract features from the original sequence data and XGBoost as a classifier to determine the subcellular localization based on the CNN’s outcome. Such kind of hybrid approaches have rarely been employed in case of the area of PCOS detection using USG images. Thus, this study has proposed an extended and novel machine learning technique where CNN architecture has been employed as the feature extractor and then stacking ensemble machine learning has been used for classification with an aim to categorize PCOS and non-PCOS USG images.

## Methodology

**Figure 1:**
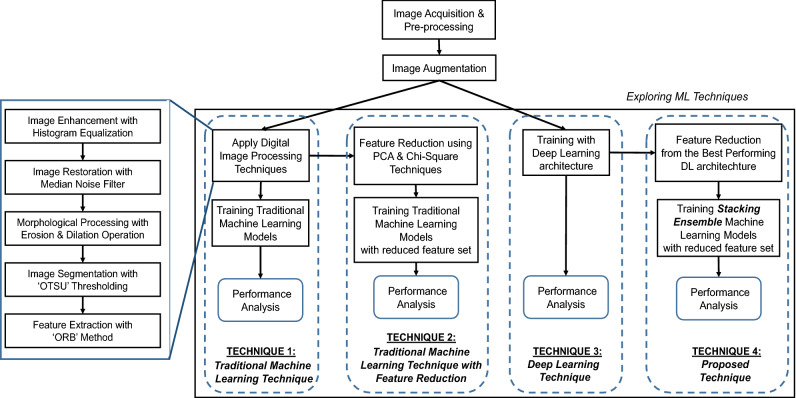
Framework of research methodology.

In this study, an extended machine learning classification technique has been proposed, trained and tested integrating ensemble ML models with Convolutional Neural Network(CNN) architecture that aims to differentiate between PCOS and non-PCOS ovaries. The ovary ultrasound images have been used as the input of the study from which the suggested method would determine whether or not the image reveals PCOS. Along with the proposed technique three other types of techniques have also been explored for classifying PCOS with an aim to conduct a comparative performance analysis of the suggested technique. The framework of the methodology used in this research is illustrated in Fig. 1. The phases involved in this research are described briefly below.

### Image acquisition and pre-processing

The images that have been utilized in this study as inputs are ultrasonography images of the ovary. There are a total of 594 images among which 123 have been acquired from various open sources from the internet and the rest of them are collected from two diagnostic centers and three hospitals of Bangladesh including Combined Military Hospital (CMH), maintaining the ethical and privacy concerns. After consulting with two radiology specialists from Mymensignh Medical College Hospital, Bangladesh and Combined Military Hospital (CMH), Bangladesh; the images have been categorized to label as PCOS and Non-PCOS for using them as training dataset. Following the data labeling, it is observed that there are 306 photos with PCOS abnormality and 288 Non-PCOS images.

Because the images have been gathered from multiple sources, their formats and sizes differed, making them unsuitable to use in predictive models. Therefore, after acquiring the images from the repository to the implementation environment, they are converted to grayscale colorspace using an OpenCV python function ‘COLOR_BGR2GRAY’ and then all the images were resized to 224X224 size. Here, the Google Colaboratory has been used as a platform for implementation incorporating mainly the python scikit-learn and TensorFlow packages. At this stage, all of the images are stored in a 3D multichannel array with the shape (594, 224, 224, 3) that contains information about the images’ plane, row, column, and channel. The labeling information or classes of each images are also stored in another 1D array which will be used as the target variable in predictive models.

### Image augmentation

Image augmentation is a powerful technique for reducing prediction error, which entails producing manipulated copies of images from the original training samples and therefore, depicting a more comprehensive set of potential data points^36^. In this research, some basic image manipulations have been applied for image augmentation which includes geometric transformations like flipping, rotating and shifting; enhancing contrast; sharpening etc. Therefore, each of the input images have been augmented to three more images at this phase.

### Applying machine learning classification techniques

Now, to train the machine learning models for classifying the images into PCOS and non-PCOS criteria; four types of technique have been conducted in this research (see Fig. 1).The first technique involves the traditional approach of ML training where after applying relevant digital image processing phases the images are trained with conventional machine learning models. In the second technique the similar steps of the previous technique have been applied but with reduced set of features extracted from the images using two types (Chi-square and PCA) of feature reduction technique. The third technique has been to train the images with deep neural network (DNN) architecture where CNN technique have been applied incorporating different types of pre-trained models for transfer learning from which the best performing DNN architechture have been explored. Finally, the proposed technique have been applied where the reduced and significant features of the images have been acquired from the best performing deep learning architecture that has been explored from the previous technique and then using those reduced set of features a stacking ensemble machine learning model have been trained to classify the PCOS and non-PCOS images.

### Performance analysis

The predictive models’ performances for each type of method are assessed on test data using several performance measures such as the Accuracy, Precision, Sensitivity (recall), Specificity, F1 score, execution time and AUC-ROC curve. Here, the execution time represents the computational time that is required for the algorithm to be trained. Other performance metrics are mainly based on the the comparative analysis of predicted values and true values of the training dataset which can be divided into four categories : true positive where the true value is positive and the predicted value is also positive; true Negative where the original value is negative, and also the predicted value has been negative; false positive in which the observation is negative, but the predicted value from the training has resulted to be positive and finally false negative that is the true value is positive, but is predicted to be negative. Basing on these evaluation the performance metrics can be written as following:

1 $$\begin{aligned} Accuracy &= \frac{True Positive + True Negative}{True Positive+ True Negative + False Positive + False Negative} \end{aligned}$$

2 $$\begin{aligned} Precision & =\frac{True Positive}{True Positive+ False Positive} \end{aligned}$$

3 $$\begin{aligned} Sensitivity\;(Recall) &=\frac{True Positive }{True Positive+ False Negative} \end{aligned}$$

4 $$\begin{aligned} Specificity &=\frac{True Negative }{True Negative+ False Positive} \end{aligned}$$

5 $$\begin{aligned} F{\text {-}}measure\;(F1score) & =\frac{2*(Precision*Sensitivity) }{Precision+Sensitivity} \end{aligned}$$

Another evaluation metrics that has been employed in this study for assessing the performances of the models is the AUC (Area Under The Curve)- ROC (Receiver Operating Characteristics) curve. It is a familiar indicator of performance for classification methods at different threshold levels where AUC stands for the level or measurement of separability, and ROC is a probability curve. The curve is typically generated based on True Positive Rate (TPR). The higher the AUC, the more the model is effective at differentiating between patients with the condition and those who do not have. d False Positive Rate(FPR).

### Ethics approval and informed consent

No human participants, human data, human tissue or any clinical data were collected for this study. However, we confirm that this research uses ultrasonography images of the ovary collected from internet and from two diagnostic centers and three hospitals of Bangladesh, maintaining the ethical and privacy concerns. Formal approval has been taken for this research work from the ethical committee (IRB) headed by the Research & Development Wing of Military Institute of Science and Technology (MIST), Bangladesh.

## Exploring machine learning techniques

The four different techniques that have been used in this study for classifying PCOS and Non-PCOS USG images are discussed hereafter.

### Technique 1: traditional machine learning technique

In this technique, after performing the digital image processing operations to the images, they were fed into several conventional machine learning models to classify them. The procedure is briefly discussed below:

#### Performing digital image processing steps

For identifying the significant areas from the images to detect the follicles, the conventional digital image processing stages have been applied to the ovary USG images (see Fig. 1: Image Processing Steps).

- The first phase is image enhancement, which is accomplished here using the histogram equalization method that improves the appearance and perception of information in pictures by providing a more consistently distributed grayscale histogram, resulting in a clearer image for viewers^37^.
- The high impulsive noises in ultrasound images create severe uncertainty causing a detrimental impact on image interpretation for clinical diagnosis^38^. Thus, here a median filter technique is used as an image restoration step for noise reduction; which is a widely utilized nonlinear filter having outstanding edge preserving qualities with ability to reduce impulsive noise^39^.
- After that, the required amount of morphological erosion and dilation has been done where dilation increases and erosion reduces the number of pixels on the edges of objects.
- The next phase is image segmentation to locate and highlight the follicles in the images for which the OTSU thresholding method has been emoloyed here. The OTSU technique is one of the most efficient automatic optimum global thresholding methods, which chooses a threshold value that maximizes the difference between pixel class variance and so divides the image into brighter and darker areas^40,41^.
- Finally, from the segmented images the key regions were extracted using Oriented FAST and Rotated BRIEF (ORB) feature extraction technique which is a faster and effective method for feature extraction to detect key points from the figure with enhanced computing efficiency as well as real-time benefits^42^ and therefore the important areas are marked in the original images.

#### Splitting into train and test data

After performing the above mentioned image processing steps the set of processed images each having size 224x224 are converted into a single dimension of using an OpenCV python function ‘FLATTEN’. The two dimentional array now contains the pixel values of 594 images where each row indicates an input image with $224*224=50176$ columns or features containing the pixel values of that image. This array is then splitted into 70% train data and 30% test data to be utilized in the machine learning models.

#### Training traditional machine learning models

Ten different machine learning models have been applied to the train and test image dataset. The prediction type here is classification since the forecasting is a binary outcome to determine whether an USG image has PCOS or not. The seven models can be categorised as classical machine learning classification models^43^ that includes Naive Bayes model, Decision Tree model, Support Vector Machine(SVM), K Nearest Neighbour(KNN) model, Naive Bayes (NB) model; bagging ensemble machine learning models^44^ including Random Forest Classifier and boosting ensemble machine learning including gradient boosting, eXtreme Gradient Boosting (XGBoost) model, Adaptive Boosting(AdaBoost) classifier and Categorical Boosting(CATBoost).

#### Ablation experiment

In machine learning, ablation commonly implies the absence of a certain element or task from an AI system. Ablation studies shed light on the relative contributions of various structural and regularization elements to the efficiency of machine learning models. An ablation experiment examines the performance of an AI system by eliminating specific components in order to determine how those components affect the system as a whole^45^. In this case, in order to assess the relative significance of image processing approaches, an ablation study has been carried out by eliminating the image pre-processing stages and running the machine learning models on the raw images.

### Technique 2: traditional machine learning technique with feature reduction

In the previous technique, it has been observed that, the input image array size was 594*X*50176 which means each row of image consists of 50176 features containing pixel values. Such enormous amount of features may cause an over-fitting problem, lowering the models’ prediction performance. In that circumstance, feature selection strategies can reduce the number of features from the dataset by selecting the most significant ones, thereby avoiding the curse of dimensionality^46^. Therefore, the second technique that has been performed in this study applied two types of feature selection approaches to find out the optimal and reduced set of features from the image dataset after conducting the image processing steps. And then those two types of dataset with reduced features are fed to the traditional machine learning models.

#### Feature reduction using PCA and Chi-square technique

The chi-square feature selection strategy, which is one of the most common and useful feature selection techniques used in machine learning, is the first feature reduction method employed here. It is a numerical analysis that estimates departure from the expected distribution and prioritizes features by studying the connection between them^47^. Another technique used for feature reduction in this study is the Principal component analysis (PCA) method, which is an efficient dimension reduction tool for feature prioritizing via quantitative simulation^48^. To achieve the feature reduction goal, PCA maps and reconstructs the original n-dimensional features to the needed k-dimensional features, where the k-dimensional features are new orthogonal features referred to as principal components^49^. Each of these strategies selected the most significant 25000 features from the 50176 features using their own methodology, resulting in two sets of datasets with reduced features at this step.

#### Training traditional machine learning models with reduced feature sets

The ten machine learning models specified in the first technique are trained again here with the dataset that have been divided into 70% train and 30% test data; where this time the models have utilized the two kinds of dataset with reduced set of features obtained by PCA and the Chi-Square feature selection techniques.

### Technique 3: deep learning technique

**Figure 2:**
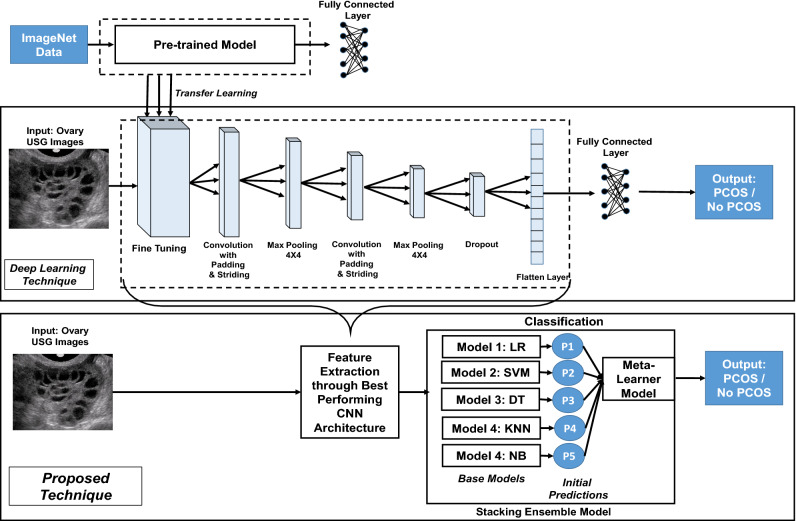
Architecture of deep learning technique and proposed technique.

After completing the image acquisition and augmentation phases, the third method performed in this study is the deep learning technique with Convolutional Neural Networks(CNN). Figure 2 depicts the deep learning architecture used here.

In this study, a modified CNN model containing multiple layers with fine-tuning have been employed to classify the USG images. The techniques used in the multiple layers of the architecture have been described hereafter.

- **Fine Tuning Layer:** The initial layer of the architecture is based on transfer learning with fine tuning that is a promising technique of machine learning which seeks to improve target learners’ performance on target domains by transferring knowledge from different but relevant domains via pre-trained models^50^. This study employs four types of best performing and popular pre-trained models, excluding their dense or fully connected layers for transfer learning; and then examines the combination of which pre-trained model in the CNN architecture can provide the best performance in case of this research. The pre-trained models that has been ulilized in this study are :
  - VGGNet16 model^51,52^ with 16 layers, 144M parameters and the best accuracy being 90.1% with Imagenet Data;
  - Xception model^53^ with 36 layers, 22M parameters and the best accuracy being 94.5% with Imagenet Data;
  - InceptionV3 model^54^ with 48 layers, 24M parameters and the best accuracy being 93.7% with Imagenet Data;
  - MobileNet model^55^ with 28 layers, 4.2M parameters and the best accuracy being 89.5% with Imagenet Data;
  However, here an ablation experiment has been conducted in this technique to assess the relative importance of transfer learning with pre-trained model for which the CNN model has been trained and tested eliminating this fine tuning layer.
- **Convolution Layer:** The following layer is a “Convolution layer” which is the CNN’s core foundational component having a set of filters or kernels with typically a smaller size than the training image, whose parameters must be trained over time and convolve with the actual image^56^. In this case, the kernel size has been considered to be 7*X*7. The convolution layer here uses padding and striding techniques to provide a more accurate result, with padding providing one extra layer to the outer picture and striding handling the space between two successive kernel positions c. In this CNN architecture the value of padding is ’same’ which implies padding the input image with zeros uniformly to the outer side; and stride is 1 which means after the convolution using the kernel the output size will be same as the input size. Now, in deep neural network, an activation function is being utilized to feed a weighted sum of input signals through, and the outcome from it is used as an input towards the following layer. In this case, ’Sigmoid’ activation function has been used which provides an output between 0 and 1 representing the probability classification outcome. The mathematical representation of the sigmoid function has been shown below:
  6 $$\begin{aligned} Sigmoid\,Activation\, Function, f(x)= \frac{1}{1+e^{-(x)}} \end{aligned}$$
  Similar kind of convolution operation has been conducted again in this CNN architecture after performing the following ’Pooling’ operation.
- **Pooing Layer:** The pooling layer is the next layer in the CNN architecture in this study which gradually reduces the spatial size of the image keeping the significant information in order to minimize the number of parameters and computations in the neural network^57^. A “Max Pooling” operation has been performed here, which yields the maximal value for each patch of the feature space with a pooling window size being 4*x*4. Similar kind of max-pooling operation has been conducted again after the second convolution operation in the architecture.
- **Dropout Layer:** The next layer is the “Dropour Layer” which helps to minimize overfitting problem as well as accelerate the training process as it changes input values to 0 randomly with a fixed frequency rate at every step throughout training phase^58^. In this CNN model, the dropout rate has been considered to be 0.5 to avoid the overfitting problem.
- **Flatten Layer** The “Flatten layer” is the subsequent layer, which turns the previous layer’s multi-dimensional output into a single dimension. A one-dimensional array is formed as a result of this layer’s output, which aids in the construction of the classifying neural network’s input layer, where the elements of the array is supplied towards each neuron. However, after performing all the previous CNN operations, the one dimensional array that has been generated in this phase possesses the most significant and reduced set of attributes from the input images. Thus, from the “Fine tuning layer” to the “Flatten layer” is considered to be the feature extraction segment of this deep learning technique.
- **Fully Connected Layer:** The “Fully Connected Layer” or “Dense Layer” is the last layer of the CNN design, which serves as the classifier layer in this technique. This layer, which is a sort of feed-forward artificial neural network, is placed at the bottom of the CNN model and within this layer, every neuron is linked to all neurons of the preceding layer, following the fundamental approach of the traditional multiple-layer perceptron neural network^59^.

As the problem in this study is a binary classification task, the CNN model has been compiled utilizing the ’binary cross-entropy’ loss function and ‘adam’ optimizer with considering ‘accuracy’ as the core evaluation metrics for each classifiers per epoch. Therefore, employing the above mentioned CNN architecture the machine learning has been executed in 30 epochs in order to train the model using the input images and provide the categorization output. However, in deep learning method there is little necessity of processing the images before training, because the internal neural network processes the image to extract prominent features for classification.

### Technique 4: proposed extended technique

This proposed technique is the combination of both deep learning approach and stacking ensemble machine learning models; where the dominant features from the input dataset have been extracted using deep learning technique’s feature extraction segment and then the stacking ensemble machine learning models are incorporated to perform the classification phase utilizing that dataset containing reduced features. The block diagram of the proposed technique has been illustrated in Fig. 2.

#### Feature extraction from best performing DL model

The internal neural networks of the deep learning architecture extract significant features from the images and the “Flattten layer” generates a single feature vector, accumulating the output of the previous layers. Therefore, after training the deep learning model with the input image dataset, the outcome obtained from flatten layer with a one dimensional array will represent the reduced set of dataset containing the vital features. As one of the core benefits of CNN architecture is that it automatically detects significant attributes from the input image performing various layer of operations without the need for human intervention or image processing; thus, the proposed technique has extracted the reduced and optimal set of features from the best performing CNN model explored from the previously explained deep learning technique in “Technique 3: deep learning technique” section.

#### Training stacked ensemble machine learning models with reduced feature set

A stacking ensemble based ML classification approach has been applied here for classifying the PCOS or non-PCOS criteria in this proposed technique that offers several advantageous perspectives, such as: (a) it analyzes heterogeneous weak classifiers as well as learns them in parallel; (b) aggregates base classifiers by training a meta-learner to produce a stronger prediction based on the forecasts of the individual weak learners; and (c) thus, it minimizes variance and also enhances predictive force of the learning process. The stacking ensemble machine learning model has been utilized as the classifier phase in this proposed technique to replace the fully connected layer in the deep learning technique. From the best performing CNN model, the reduced set of features has been explored and then utilized as the input training data for the stacked machine learning classifiers.

Here, in the proposed stacked ensemble model, the dataset with reduced feature set is initially sent to the base learners. At this phase, the five types of widely utilized traditional machine learning classifiers have been considered to be the weak learners or base classifiers at level 0 of the stacked model, which are: Logistic Regression(LR), Support Vector Machine(SVM), Decision Tree(DT), K-Nearest Neighbour(KNN) and Gaussian Naive Bayes(NB) classifiers. Each of these base models gets trained independently using their respective prediction algorithms, resulting in forecasts symbolized p1, p2, p3, p4, and p5 in Fig. 2. Following that, the predictions acquired from the level 0 models are fed into level 1 where a single classification model, or meta-learner, learns to generate final prediction from it. The meta-learner has been created at level 1 employing one stronger machine learning classifier. Here, while keeping the same base models at level 0, five types of classifiers have been explored as meta learners in level 1 which resulted in five varieties of the proposed architecture with an aim to explore the best performing one. The meta learner that has been used here is one of the five types of bagging or boosting classifiers ( Random Forest, Adaptive Boosting, Gradient Boosting, CAT Boosting and XGBoosting) that is ultimately trained on top of level 0 to generate the final output based on the predictions returned by the base models. Thus, an extended stacked ensemble ML classifier has been proposed, trained and evaluated incorporating five types of traditional classifiers as base models and one boosting or bagging type of classifier as meta learner with an aim to differentiate between PCOS and non PCOS patient ovary USG images. The pseudocode of the proposed technique for PCOS detection has been shown in Algorithm 1.
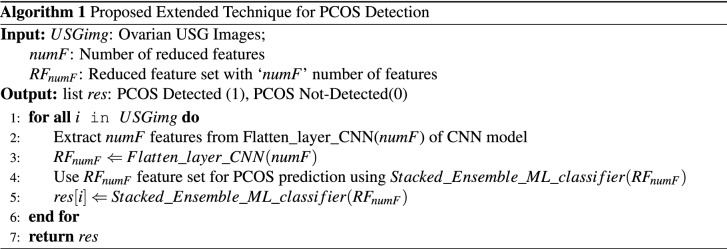


## Result analysis

The following sections have been organized to present the findings from the interpretation of research results.

**Table 1:** Performance analysis of test data using traditional machine learning technique with and without image pre-processing (Technique 1).

ML models	Without image pre-processing						With image pre-processing					
	Accu.	Prec	Rec.	Spec	F1-s	Time	Accu.	Prec.	Rec.	Spec	F1-s	Time
Logistic regression	0.739	0.739	0.74	0.73	0.74	5.85	0.756	0.754	0.76	0.74	0.76	4.3
SVM	0.762	0.762	0.76	0.76	0.74	370.7	0.807	0.808	0.81	0.79	0.80	268.2
Decision tree	0.714	0.715	0.72	0.71	0.71	24.8	0.737	0.773	0.73	0.74	0.75	10.4
KNN	0.718	0.719	0.72	0.69	0.72	13.9	0.849	0.870	0.85	0.84	0.85	9.1
Naive Bayes	0.714	0.716	0.71	0.70	0.72	6.3	0.760	0.761	0.77	0.76	0.76	4.2
Random forest	0.778	0.779	0.77	0.74	0.77	8.2	0.883	0.883	0.88	0.87	0.88	5.8
Gradient boosting	0.791	0.792	0.80	0.79	0.82	44.3	0.855	0.856	0.86	0.86	0.85	31.8
Adaptive boosting	0.718	0.716	0.71	0.72	0.71	272.2	0.815	0.813	0.82	0.81	0.81	218.9
XG boosting	0.806	0.810	0.80	0.79	0.81	172.2	0.856	0.855	0.85	0.86	0.86	150.1
CATBoost	0.712	0.710	0.72	0.71	0.70	72.2	0.790	0.792	0.79	0.78	0.79	74.1

**Figure 3:**
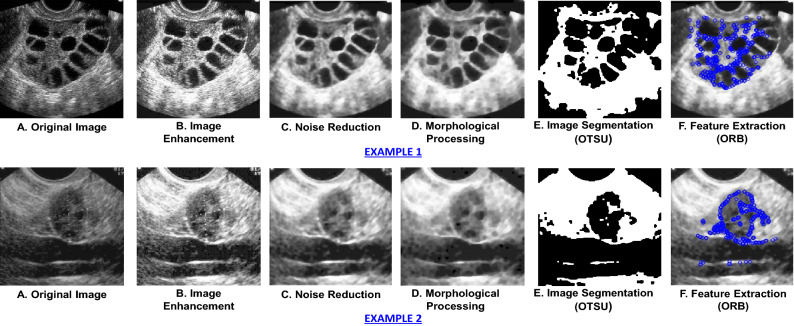
USG scans with image processing steps.

### Findings from machine learning techniques

Employing technique 1, the performances of the ten types of traditional machine learning models using several performance metrices and their execution times have been illustrated in Table 1. In this technique, after image acquisition and augmentation phases, all the images are being processed using image processing techniques. The examples of USG scan with image processing technique is shown in Fig. 3. The figure makes it apparent that image pre-processing steps change the original images substantially clearer while also allowing for the identification of the ovarian cysts that were barely apparent in the original image in the impacted locations. The models are then trained using the enhanced pictures with feature extraction containing 50176 features of pixel values, which have been collected at the completion of image processing steps. Here, for conducting ablation study to analyze the relative importance of image processing steps, the models have been trained and tested firstly without image pre-processing and then with image pre-processing. From the performance analysis in Table 1, it can be observed that, for all the classifiers, the performances significantly enhances after applying image-preprocessing steps. In case of training the models without image pre-processing, the best performance has been acquired in XGBoosting model with 80.6% accuracy. On the other hand, employing image pre-processing techniques, Random Forest classification model outperforms all other classifiers in terms of performing with highest accuracy (88.3%) in less computation time (5.8 seconds) whereas the Naive Bayes classifier provides the least accuracy (76.0%) and SVM classifier takes the highest execution time (368.2 seconds). The AUC-ROC curve of the analysis without image pre-processing and with image pre-processing has been shown in Fig. 6a,b. Thus, the performance analysis clearly reveals that, image pre-processing techniques plays a significant role in acquiring good performances with machine learning classifiers.

In the second type of technique, the two datasets with reduced features of 25,000 pixel columns acquired from the two types of feature reduction algorithms are applied to the traditional machine learning classifiers. The performances and execution times of the ten types of traditional machine learning models with both type of feature sets are shown in Table 2. From the table, it is apparent that after feature reduction the execution time for each of the models reduces significantly. It can be also observed that, most of the models perform better when employing chi-square feature sets than PCA technique. However, the Random Forest classifier outperforms the others, with an accuracy of 88.8% using the chi-square approach and Gradient Boosting model performs comparatively better using PCA feature set with 80.5% accuracy.

**Table 2:** Performance analysis of test data using traditional machine learning technique with feature reduction (Technique 2).

ML models	Using Chi-square feature set						Using PCA feature set					
	Accu.	Prec	Rec.	Spec	F1-s	Time	Accu.	Prec.	Rec.	Spec	F1-s	Time
Logistic Regression	0.768	0.769	0.76	0.77	0.77	3.7	0.760	0.761	0.76	0.76	0.76	3.9
SVM	0.823	0.820	0.82	0.82	0.82	111.9	0.776	0.770	0.77	0.77	0.77	113.9
Decision Tree	0.639	0.647	0.64	0.69	0.63	14.6	0.637	0.673	0.63	0.54	0.65	10.2
KNN	0.778	0.822	0.73	0.67	0.72	6.3	0.782	0.819	0.77	0.72	0.77	7.6
Naive Bayes	0.698	0.699	0.69	0.69	0.69	2.75	0.697	0.664	0.67	0.65	0.66	3.3
Random Forest	0.888	0.886	0.89	0.89	0.88	4.3	0.793	0.781	0.72	0.70	0.72	4.8
Gradient Boosting	0.853	0.859	0.86	0.86	0.85	35.2	0.805	0.809	0.81	0.81	0.80	36.8
Adaptive Boosting	0.773	0.789	0.77	0.75	0.77	177.2	0.790	0.792	0.79	0.78	0.79	104.1
XG Boosting	0.783	0.779	0.77	0.74	0.77	75.2	0.765	0.769	0.76	0.74	0.76	86.8
CAT Boost	0.811	0.813	0.81	0.80	0.81	48.3	0.799	0.795	0.79	0.79	0.80	50.5

**Table 3:** Performance analysis of test data using deep learning technique (Technique 3).

Deep learning models	Accuracy	Precision	Recall	Specificity	F1-Score	Time (s)
CNN without transfer learning	0.7479	0.75	0.78	0.74	0.75	45.9
CNN with VGGNet16	0.9780	0.97	0.96	0.96	0.97	82.6
CNN with Xception	0.9187	0.92	0.91	0.92	0.91	96.7
CNN with InceptionV3	0.7625	0.76	0.75	0.76	0.76	55.6
CNN with MobileNet	0.9162	0.91	0.92	0.91	0.91	70.8

**Figure 4:**
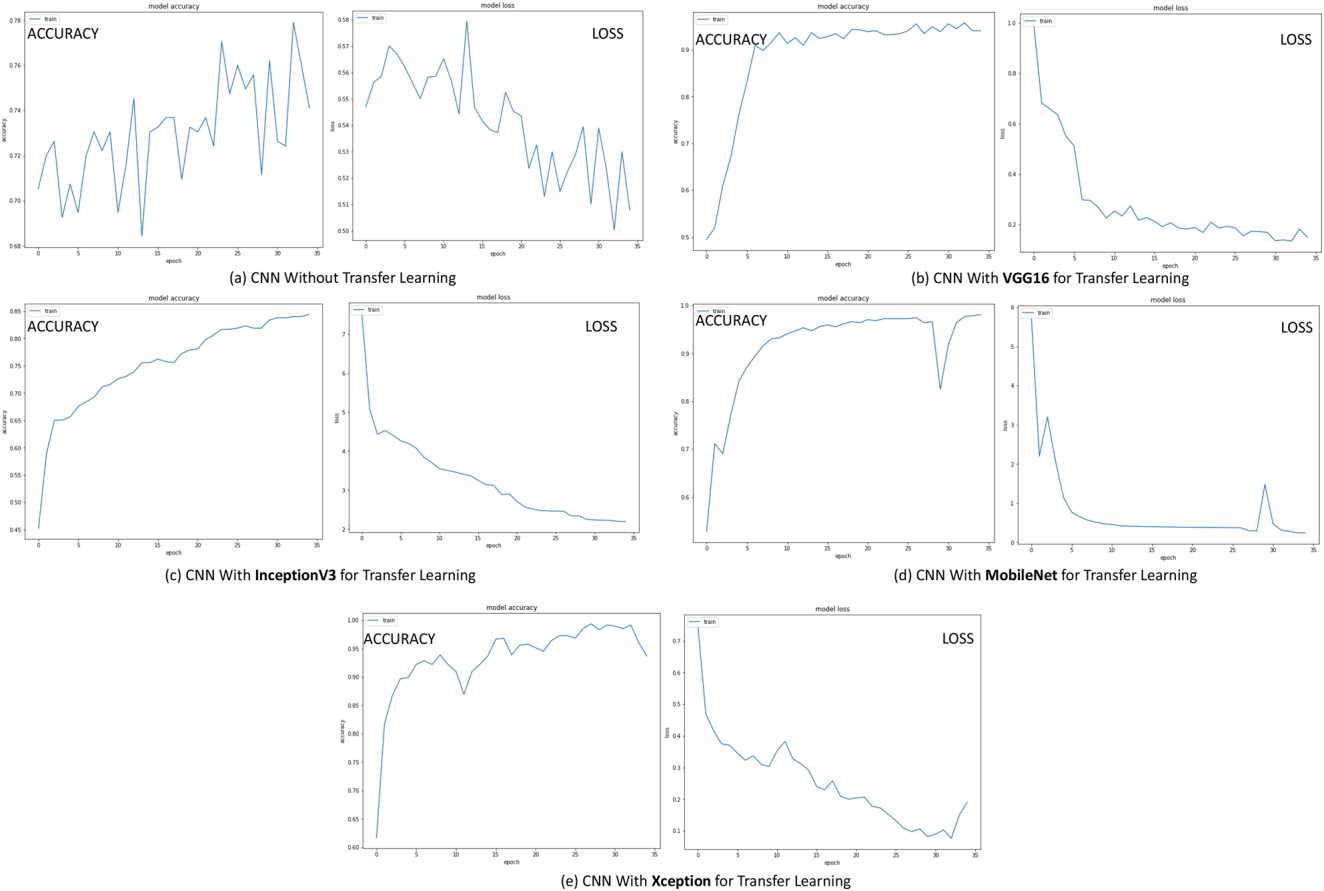
Accuracy and Loss per epoch for DNN models (technique 3) (**a**) CNN without transfer learning (**b**) CNN with VGGNet16 for transfer learning (**c**) with InceptionV3 for transfer learning (**d**) with MobileNet for transfer learning (**e**) with Xception for transfer learning.

The 3rd technique has followed deep learning architecture in which a variety of pre-trained model has been employed in the initial layer using the transfer learning (TL) approach. For analyzing the relative importance of this transfer learning layer, an ablation study has also been conducted in this phase where the CNN model has been trained and tested without the transfer learning layer. Each model has been trained using 30 epochs. Table 3 shows the performances of the deep learning technique on a test dataset without transfer learning layer as well as utilizing four different types of pre-trained models each one executing over 30 epochs. Figure 4 shows the accuracy and loss per epoch for each type of the dnn techniques. Figure 6c illustrates the AUC-ROC curve employing various forms of DNN technique. Here, from the performance analysis it is evident that all the deep learning approaches have gained significantly higher accuracy with the “VGGNet16” pre-trained model surpassing the others, obtaining 97.80% accuracy in 82.7 seconds execution time. It is also noticeable that, the CNN model without transfer learning layer performs much worse with only 74.79% accuracy which is much lesser than the models employing transfer learning. This analysis reveals that, transfer learning plays a key role in efficient prediction of the classes. However, the performance analysis from 3 also shows that though the accuracy of the models using this dnn technique is much higher but the execution time for prediction is also very high which can be considered as a drawback of this technique.

**Table 4:** Performance analysis of test data using proposed technique (Technique 4).

	Base Models	Meta-learner	Accuracy	Precision	Recall	Specificity	F1-score	Time (s)
Feature Extraction	LR	Random Forest	0.9844	0.98	0.98	0.97	0.98	0.09
with CNN	SVM	XGBoost	0.9989	0.99	1.00	1.00	0.99	0.05
& Classification	DT	AdaBoost	0.9959	0.99	0.98	0.99	0.99	1.07
with Stacking	KNN	GradBoost	0.9922	0.99	1.00	0.99	0.99	0.09
Ensemble Model	NB	CATBoost	0.9958	1.0	0.99	0.99	1.0	1.12

**Figure 5:**
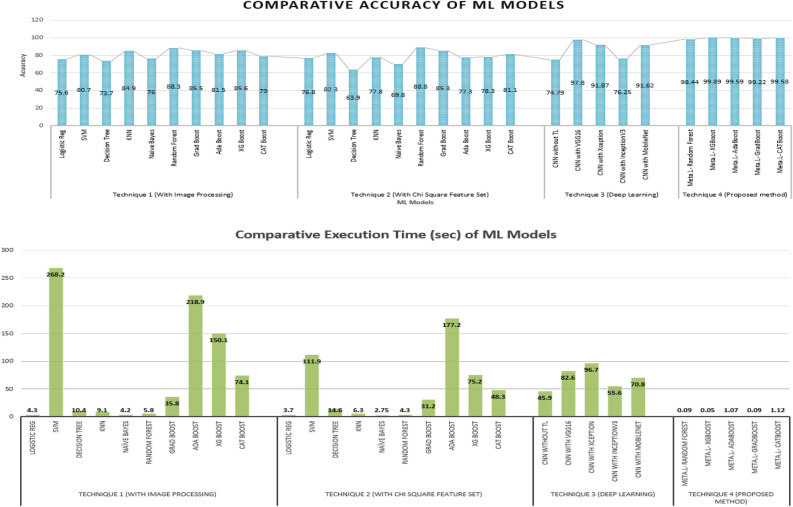
Comparative analysis of (**a**) accuracy and (**b**) execution time for ML models using different techniques.

**Figure 6:**
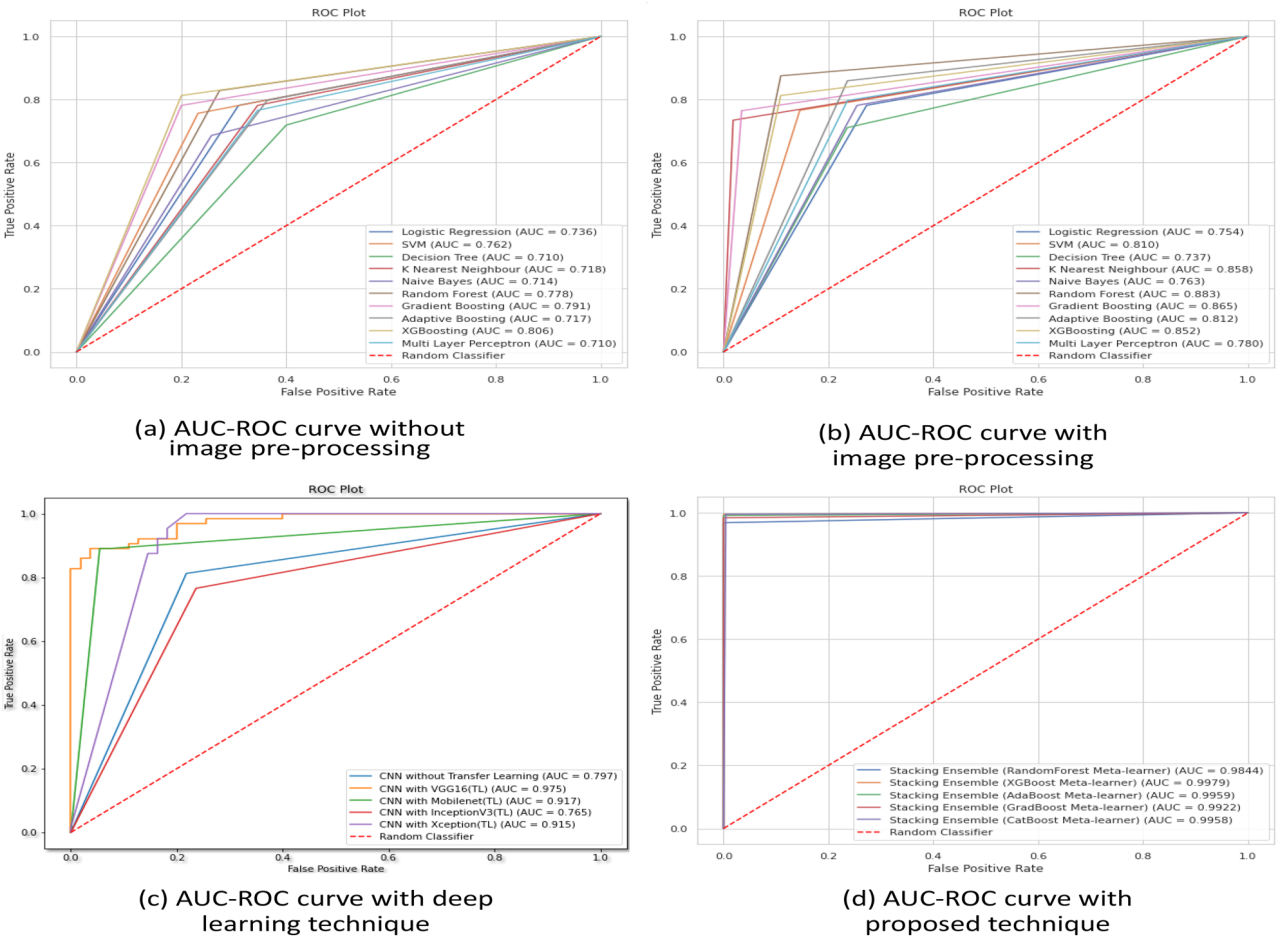
AUC-ROC curve for ML models (**a**) without image pre-processing (technique 1) (**b**) with image pre-processing (technique 1) (**c**) with deep learning (technique 3) (**d**) with proposed technique (technique 4).

Finally, in the proposed extended technique, the input dataset for the machine learning classifiers is the flatten layer’s output feature vector from the deep learning architecture incorporating the best performing CNN architecture with “VGGNet16” pre-trained model used for tranfer learning. The extracted dataset with reduced features now contains only the 32 most significant attributes from the USG ovary images gained by the deep learning method’s neural networks. This dataset with extracted features has been fed to the proposed stacking machine learning classifiers where all the stacked ensemble model contains same base learners but five different types of meta-learners and therefore their comparative predictive performances are analyzed using performance metrics, as shown in Table 4. The results reveal that, the performances of all of the models have improved dramatically, with the XGBoost classification model as meta-learner achieving the greatest accuracy of 99.89% in just 0.05 seconds of execution time. Here, from this analysis it is apparent that, for all the varieties of the proposed technique the accuracy is always more than at least 98% with comparatively very less execution times. The AUC-ROC curve of the proposed technique has been shown in Fig. 6d, which also shows significantly efficient AUC scores than the other types of predictive models.

However, here the best performing meta-learner “XGBoost” classifier is an efficient open-source boosting type of ensemble machine learning model. The model is trained here by minimizing the loss of an ‘objective’ function against the dataset with an aim to minimize the error, where the appropriate choice of loss function in XGBoost model is a critical hyperparameter to acquire desired performances. As the problem in this article is a binary classification task with two class labels, thus in the proposed technique the loss function used for predicting probabilities is ‘objective=binary:logistic’.

### Comparative analysis

The comparative performance analysis of the accuracy and execution times of various machine learning models employing the four types of techniques in this study for PCOS detection are represented graphically in Fig. 5. Also, the AUC-ROC curve of the techniques have been illustrated in Fig. 6. The comparative analysis of the four methods for detecting PCOS from ultrasound images using machine learning techniques reveals that, the performances of the ten types of ML models employing traditional approach in technique 1 with all input features from the images are much poorer in terms of all the performance metrics (see Table 1). Though the performances of the models enhances after applying image pre-processing steps, but still the highest performance can be achieved in this technique with an accuracy of 88.3% in 5.8sec execution time employing Random Forest classifier. The performances of the models enhance a little bit when numerical feature selection techniques like chi-aquare and PCA have been applied to the dataset in technique 2, where the highest accuracy has been gained to be 88.8% with 4.3 sec execution time with Random Forest classifier trained with reduced feature set of chi-square technique (see Table 2).

However, the performances of detecting the PCOS get much higher when deep learning model is utilized for classification in technique 3 employing transfer learning technique, with an highest accuracy of 97.80% with VGGNet16 used as the layer of transfer learning in the proposed CNN architecture (see Table 3). But, the limitations of using deep learning models are that they are expensive to train and demand a lot of processing power and time. Therefore, as compared to typical machine learning models the execution time is significantly longer in deep learning models, which is evident in Table 3 (best performing model takes 82.6 sec execution time). To overcome these drawbacks, this study have implemented a hybrid model in method 4, in which the feature extraction phase from the images has be conducted by the best performing deep learning model and then the stacking ensemble machine learning models are utilized to do the classification. This method yields substantially higher accuracy in a short amount of time (see Table 4). It is noticeable that, for each of the ML model the accuracy increases and execution time decreases dramatically with the proposed technique. For example, for Random Forest classifier the best acquired accuracy with technique 1 is 88.3% and with technique 2 is 88.8%; which enhances significantly providing 98.44% accuracy while employing as a meta-learner of the stacked ensemble model of the proposed technique. Also, as the classifiers predicts the classes based on only 32 dominant features of the USG images, the execution times are comparatively much lesser in the proposed technique. Therefore, the comparative performance analysis reveals that, the proposed technique employing CNN with “VGGNet16” pre-trained model used for transfer learning as the feature extractor and the stacking ensemble model with “XGBoost” classification model used for meta-learner as the classifier of this problem can categorize PCOS and non-PCOS USG images in not only with the best performances but also solve the problem in least execution time.

## Discussion and conclusion

The presented work in this research can be a pioneer study in the domain of PCOS detection which has incorporated the advantages of both conventional and deep learning techniques. The findings of this study can be significantly beneficial towards both patients and healthcare providers in identifying PCOS quickly and efficiently. The suggested hybrid method has combined transfer learning technique using powerful pre-trained models in a CNN architecture to extract the most significant feature set from the images and then stacking ensemble machine learning technique have been employed to classify the images. To analyze the efficacy of the model and evaluate its performances, three types of other existing methodologies have also been performed in this study. The first two existing techniques for PCOS detection from USG images using traditional ML algorithms have appeared to be much inefficient as they provides significantly less accuracy as well necessitate tedious digital image processing steps for feature extraction and also requires another feature selection method for the reduction of massive amount of image features. Also, the third existing technique with only deep learning technique requires much higher computational space and time. Ultimately, after several analysis the experimental findings reveal that, the proposed hybrid strategy of employing the “VGG16” pre-trained model for transfer learning in the CNN architecture for feature extraction and then the “XGBoost” machine learning model as the meta-learner of stacking ensemble model for image classification yields the maximum accuracy of 99.89% with a relatively shortest execution time to detect PCOS from ultrasound images.

An effective machine learning approach to detect PCOS from ovarian ultrasound images is considered to be a potentially valuable solution for recovering thousands of women’s reproductive health. Most of the studies that had been conducted in this field (for example the works conducted in^15–26^) for PCOS detection are the implementation of various forms of digital image processing techniques for follicle detection and then applying conventional machine learning models for classification. Recently, a few researchers have also focused on applying deep neural network with CNN architecture to detect PCOS from medical images (for example the works conducted in^30,31^). Yet, little or no research has been conducted in this area for PCOS diagnosis that uses both traditional ML classifiers and deep neural networks together with transfer learning and fine tuning. Moreover, even if some other research from different domains have adopted this type of integrated model (for example^35^), but hardly any study has been found that combined the CNN techniques with stacked ensemble machine learning classifiers with an aim to detect anomaly from medical images. Thus, to the best of our knowledge, the proposed technique in this study, where CNN with transfer learning and fine tuning utilized as the feature extractor and then stacked ensemble machine learning with the integration of classical and boosting ensemble models as the classifier is a unique and novel solution in the domain of medical image analysis for disease identification.

The findings of this study will greatly benefit healthcare practitioners in identifying PCOS using ultrasound images more quickly and accurately combining the advantages of both traditional machine learning and deep learning approaches and thus it is anticipated to be widely used in the real-world scenario. However, one of the study’s limitations was that, collecting medical images of the patients in the least developing country is quite challenging and as a result the machine learning techniques have been applied on a limited number of images (594 images) due to the lack of a dataset. Additionally, the proposed technique is based on deep learning feature extraction and stacked ensemble machine learning classifier which is quite difficult to make explainable and thus may seem like black box towards the physicians. Therefore, in the future, the researchers look forward to discover more about PCOS identification employing larger datasets, incorporating the concepts of explainable AI (XAI) and federated learning as well as use similar technique to detect other clinical disorders.

## Data Availability

The dataset generated and analyzed during the current study are not publicly available due to the fact that we are still using it for another publication, but are available from the corresponding author on reasonable request.
